# Artificial Intelligence in Rare Diseases: Workflow-Integrated Precision Kidney Care

**DOI:** 10.3390/clinpract16060101

**Published:** 2026-05-27

**Authors:** Charat Thongprayoon, Francesco Pesce, Wisit Cheungpasitporn

**Affiliations:** 1 Division of Nephrology and Hypertension, Department of Medicine, Mayo Clinic, Rochester, MN 55905, USA; charat.thongprayoon@gmail.com; 2 Division of Renal Medicine, Ospedale Isola Tiberina-Gemelli Isola, 00168 Rome, Italy; francesco.pesce2@unicatt.it; 3 Department of Translational Medicine and Surgery, Università Cattolica del Sacro Cuore, 00168 Rome, Italy

**Keywords:** rare diseases, artificial intelligence, genomic diagnosis, clinical decision support, nephrology

## Abstract

Rare diseases affect over 300 million individuals worldwide yet remain underdiagnosed and poorly characterized due to fragmented data, small cohorts, and phenotypic heterogeneity. Advances in artificial intelligence (AI) are enabling integration of genomics, imaging, electronic health records, and patient-generated data to support diagnosis, phenotyping, prognosis, and therapeutic discovery. In kidney care, these capabilities are reflected in tools for genomic variant prioritization, AI-assisted histopathology, and integrated risk stratification models for rare and complex kidney diseases. This review synthesizes current AI applications across the rare disease continuum and proposes a clinically grounded framework to distinguish exploratory models from systems that are methodologically robust and operationally deployable. We highlight advances that address data sparsity and heterogeneity, alongside persistent challenges in validation, generalizability, equity, and workflow integration. Finally, we outline future directions, including federated learning, digital twins, and AI-driven clinical decision agents, as pathways toward precision-guided, workflow-integrated rare disease care.

## 1. The Rare Disease Paradox and the Diagnostic Odyssey

Rare diseases are typically defined based on low prevalence thresholds, although definitions vary by region [1,2,3,4]. The European Medicines Agency defines a rare disease as one affecting fewer than 5 in 10,000 individuals, while broader international frameworks increasingly recognize the combination of low prevalence, clinical complexity, and substantial unmet medical need [1,2,3,4]. Despite their individual rarity, rare diseases collectively affect more than 300 million individuals worldwide, representing a substantial but often underrecognized global health burden [1,2,3,4].

Patients frequently endure years of sequential referrals, misdiagnoses, and delayed genetic testing before receiving a definitive diagnosis [5,6,7,8]. This prolonged diagnostic odyssey, often spanning several years, reflects a systemic diagnostic delay marked by fragmented care pathways, repeated incorrect diagnoses, and delayed access to definitive genomic evaluation. Importantly, this delay is not merely inefficient but clinically harmful, as it postpones disease-modifying interventions and compounds psychological and financial burdens [9,10,11,12].

Traditional statistical and machine-learning paradigms struggle in this setting [13,14]. Rare diseases violate core assumptions of population-level modeling due to small sample sizes, heterogeneous phenotypes, and long disease trajectories [15,16,17,18,19]. This creates a fundamental mismatch between conventional evidence-generation frameworks, which prioritize population-level inference, and the realities of rare disease care, which are inherently individualized. Given the inherent data sparsity and heterogeneity of rare diseases, multiple complementary AI paradigms have emerged, each addressing distinct methodological constraints (Table 1) [13,16,20,21,22,23,24,25,26]. Consequently, clinical decision-making in rare diseases often incorporates individualized N-of-1 reasoning. AI systems designed for this domain should therefore move beyond population-level prediction toward individualized, mechanism-informed reasoning, with a particular emphasis on compressing the diagnostic timeline described above. In this context, AI-enabled genomic diagnostics represent a key application, offering the potential to reduce selected components of diagnostic delay from months or years to shorter clinically meaningful intervals in optimized settings [27,28,29,30,31].

This manuscript is structured as a narrative review aimed at synthesizing current and emerging applications of artificial intelligence in rare diseases, with a focus on clinical translation in kidney care. A targeted literature search was conducted using PubMed, Embase, and Google Scholar to identify relevant publications from January 2015 through January 2026. Search terms included combinations of ‘rare disease,’ ‘artificial intelligence,’ ‘machine learning,’ ‘genomics,’ ‘federated learning,’ ‘digital twins,’ ‘clinical decision support,’ and ‘precision medicine.’ Additional references were identified through citation tracking of key articles.

Study selection was guided by relevance to rare disease diagnosis, phenotyping, prognosis, and therapeutic decision-making, with emphasis on methodological rigor, translational applicability, and representation of key AI paradigms. Priority was given to peer-reviewed original studies, high-impact reviews, and representative methodological papers that illustrate clinically meaningful applications or emerging frameworks. Given the scope of the review, formal inclusion and exclusion criteria were not applied in a systematic manner; instead, selection was purposive to ensure coverage of both established and evolving approaches relevant to rare disease care.

Evidence was synthesized using a concept-driven approach, integrating findings across domains including genomics, federated learning, and digital twin modeling to construct a clinically grounded evaluative framework. Emerging areas, such as agentic AI systems, are included to provide forward-looking context and are explicitly distinguished from empirically validated applications. While this approach allows for flexible integration of a rapidly evolving literature, it may introduce selection bias and does not provide a comprehensive systematic appraisal of all available studies.

## 2. AI-Accelerated Genomic Diagnosis: Compressing Diagnostic Timelines

### 2.1. Core Concept

As outlined in Section 1, patients with rare diseases often experience prolonged diagnostic delays spanning several years, driven by fragmented clinical data, phenotypic heterogeneity, and limited access to specialized expertise. AI-enabled genomic diagnostics directly address this challenge by restructuring the diagnostic process, shifting from sequential, trial-and-error evaluation toward integrated, data-driven prioritization. Central to this approach is the use of structured phenotype representations. Among available ontologies, the Human Phenotype Ontology (HPO) is prioritized because it provides a granular, hierarchical, and computationally tractable framework specifically designed to capture phenotypic abnormalities and enable genotype–phenotype matching [6,31,32,33].

Unlike broader clinical terminologies such as the Systematized Nomenclature of Medicine—Clinical Terms (SNOMED CT), which are optimized for clinical documentation and interoperability, or curated gene–disease resources such as Online Mendelian Inheritance in Man (OMIM), which primarily catalog genetic associations, HPO is structured to support phenotype-driven diagnostic reasoning. Its standardized vocabulary and hierarchical relationships allow efficient encoding of patient-specific features and facilitate algorithmic comparison with known disease phenotypes, making it particularly well suited for AI-enabled variant prioritization in rare disease contexts.

AI-enabled variant prioritization, when coupled with deep phenotype matching using standardized ontologies such as the Human Phenotype Ontology (HPO), has the potential to improve diagnostic efficiency and shorten components of the diagnostic process. Rather than representing solely incremental efficiency gains, these approaches may support more rapid and hypothesis-driven diagnostic workflows in selected clinical settings. The clinical objective is to reduce diagnostic delays while maintaining clinical rigor and appropriate expert oversight [34].

### 2.2. Architecture of a High-Sensitivity Diagnostic Pipeline

A clinically viable pipeline integrates multiple AI components, each optimized for a specific task: [21,28,35,36,37,38,39,40,41,42,43,44,45].

1. DeepVariant for deep-learning–based variant calling with high fidelity.
2. SpliceAI for detection of cryptic and non-canonical splice variants.
3. AlphaMissense and REVEL for probabilistic assessment of missense pathogenicity.
4. Phenotype-aware ranking tools (e.g., Exomiser) that align genetic variants with patient-specific HPO profiles.

**Figure 1:**
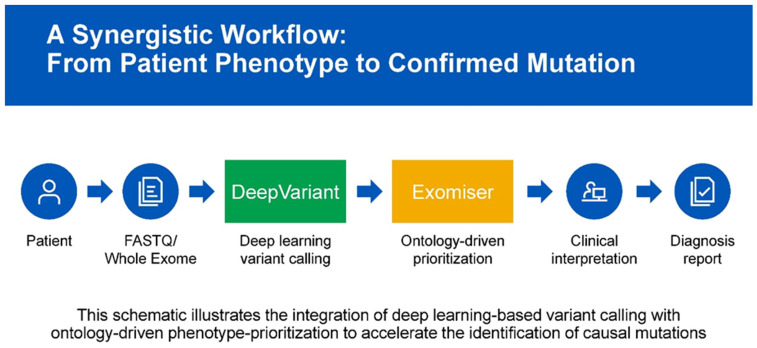
AI-enabled genomic diagnostic workflow from patient phenotype to confirmed causal mutation. Clinical evaluation initiates whole-exome sequencing, generating raw sequencing data (FASTQ). Deep learning–based variant calling (e.g., DeepVariant) identifies genomic variants with high sensitivity. Ontology-driven prioritization tools (e.g., Exomiser) integrate patient-specific phenotypes using structured vocabularies such as the Human Phenotype Ontology, which enables precise phenotype encoding and computational matching to known genotype–phenotype relationships. Shortlisted variants undergo expert clinical interpretation, culminating in a structured diagnostic report. This integrated pipeline demonstrates how artificial intelligence compresses the diagnostic timeline by combining high-fidelity variant detection with phenotype-aware ranking, enabling efficient identification of causal mutations in rare diseases.

Variants are systematically filtered and ranked against the patient’s phenotype, producing a concise shortlist suitable for expert clinical review. Importantly, the clinical objective is not exhaustive variant identification but efficient prioritization of biologically plausible candidates that directly address the diagnostic delays described in Section 1. A clinically deployable architecture integrating deep learning–based variant calling with phenotype-aware prioritization is illustrated in Figure 1.

While AI-enabled genomic pipelines have demonstrated improvements in the efficiency of variant prioritization, their real-world performance remains variable and context-dependent [21,28,35,36,37,38,39,40,41,42,43,44,45]. Reported sensitivity and diagnostic yield are influenced by multiple factors, including sequencing quality, completeness and accuracy of phenotypic annotation, and the availability and curation of reference databases. In clinical practice, false positive burden represents a significant challenge, particularly when large numbers of candidate variants are generated and require expert review and adjudication [21,28,35,36,37,38,39,40,41,42,43,44,45]. In addition, model performance may be reduced in populations that are underrepresented in training datasets, limiting generalizability and raising concerns regarding bias and equity. Furthermore, variability across sequencing platforms and institutional workflows may affect reproducibility and consistency of results. Collectively, these limitations underscore the need for rigorous external validation, careful clinical integration, and continued reliance on expert interpretation when applying AI-assisted genomic pipelines in rare disease diagnostics.

Recent advances in large-scale genomics foundation models further extend this pipeline from variant prioritization toward mechanistic hypothesis generation and biological interpretation. AlphaGenome [46,47], a sequence-to-biology model developed by Google DeepMind, directly processes raw DNA sequence to predict regulatory and molecular effects across both coding and noncoding regions, including gene expression, splicing, chromatin accessibility, transcription factor binding, and long-range genomic interactions [48]. Unlike modular pipelines that rely on independent, task-specific models, AlphaGenome provides a unified representation of genomic function, enabling context-aware interpretation of variants beyond protein-coding regions. In practice, an optimized clinical workflow may pair conventional pipelines for high-sensitivity variant detection and filtering with foundation models for downstream mechanistic explanation, reframing the central clinical question from “Is this variant pathogenic?” to “How does this variant alter biological function?” This complementary integration represents a shift from probabilistic ranking toward biology-informed mechanistic interpretation, while remaining investigational and requiring expert clinical interpretation and external validation before routine clinical deployment in rare disease genomics [46,47] (Table 2).

However, these integrated models also introduce important limitations. Their deep learning architectures often reduce interpretability, making it challenging to trace specific predictions to biologically transparent mechanisms. In addition, their performance depends heavily on large-scale training datasets, which may not fully represent diverse populations or rare variant distributions, raising concerns regarding bias and equity. Furthermore, clinical generalization remains an ongoing challenge, as model performance may vary across sequencing platforms, patient populations, and healthcare settings. These limitations underscore the need for rigorous external validation, careful clinical integration, and continued human oversight when deploying such models in rare disease diagnostics.

### 2.3. Validation Metrics That Matter

In rare disease diagnostics, high sensitivity is particularly important. The clinical cost of missing a pathogenic variant far outweighs the burden of reviewing false positives. Accordingly, pipelines should target: [44,45,49,50,51,52,53,54].

• Sensitivity approaching or exceeding 90 percent for known pathogenic variants
• Top-3 or Top-5 recall rates exceeding 80 percent in phenotype-driven prioritization tasks
• Median end-to-end turnaround times of ≤7 days in clinically optimized settings

These thresholds should be interpreted as aspirational and context-dependent benchmarks rather than universally established standards, as performance varies across datasets, sequencing platforms, patient populations, and implementation environments.

Precision optimization should occur only after sensitivity thresholds are met. This hierarchy of metrics reflects clinical reality rather than abstract model performance.

### 2.4. Real-World Nephrogenetics Implementation

Published nephrogenetics workflows integrating genomic sequencing with phenotype-aware prioritization have demonstrated high diagnostic yield and the potential to improve diagnostic efficiency in selected clinical settings [55,56,57,58,59]. Across reported cohorts, AI-assisted approaches that combine variant calling, splice prediction, and phenotype-driven ranking with expert clinical interpretation have improved the efficiency of variant prioritization and diagnostic workflows. However, these improvements are context-dependent and have been most consistently observed in specialized centers with curated datasets and established genomic infrastructure. Reported sensitivity, diagnostic yield, and turnaround times vary across studies and are influenced by cohort characteristics, sequencing strategies, and validation frameworks, and may not be generalizable to broader clinical environments.

Rather than representing a single standardized pipeline, these approaches reflect a converging model of care in which artificial intelligence augments, but does not replace, expert clinical judgment. In practice, the clinical impact arises from improved triaging of candidate variants, reduction in manual review burden, and earlier identification of plausible diagnoses within existing laboratory and clinical infrastructures [44,45].

For example, a young adult presenting with unexplained chronic kidney disease, persistent microscopic hematuria, and a family history of renal disease may undergo whole-exome sequencing. In a conventional workflow, interpretation of variants of uncertain significance can be time-intensive and inconclusive. Using AI-assisted variant prioritization integrated with structured phenotype encoding (e.g., Human Phenotype Ontology terms), candidate variants in genes such as *COL4A3* or *COL4A5* may be prioritized based on phenotype–genotype concordance. This can support earlier diagnostic consideration of Alport syndrome, facilitating timely initiation of renin–angiotensin system blockade and appropriate family screening. While such workflows require expert validation, they illustrate how artificial intelligence can enhance efficiency and support clinical decision-making in nephrogenetics.

Overall, current evidence supports the feasibility of AI-augmented genomic diagnosis in rare nephropathies and suggests that, when implemented within supervised clinical workflows, such systems can accelerate specific components of the diagnostic process [49,50,54,55]. However, these gains are not uniform, and broader generalizability and reproducibility remain dependent on external validation, prospective evaluation, and integration into routine nephrology practice [60,61]. As such, AI-assisted diagnostic support should be interpreted as a promising but evolving capability rather than an established standard of care. The critical next step is translation into routine nephrology workflows, where AI-enabled genomic pipelines can be systematically embedded into clinical decision-making processes; several high-yield, workflow-integrated use cases are outlined in Table 3.

## 3. Beyond Single Centers: Federated Learning for Rare Disease Discovery

### 3.1. The Limits of Local Excellence

Even the most advanced single-institution pipelines cannot overcome the fundamental limitation of ultra-rare disease prevalence [62,63,64,65]. Discovery of novel gene–disease associations, progression models, and therapeutic biomarkers requires learning across institutions [66,67].

### 3.2. Federated Learning as a Privacy-Preserving Solution

Federated learning enables distributed model training without sharing patient-level data [66,68,69,70,71]. Each institution trains a local model on harmonized data, and model updates or parameters, often combined with privacy-enhancing safeguards, are aggregated centrally rather than transferring raw patient-level data. This paradigm preserves privacy while enabling collective intelligence across institutions [66]. However, reproducibility and real-world performance remain important considerations, as model outputs may vary across sites due to differences in data structure, coding practices, and patient populations, even when harmonized training protocols are applied.

As an illustrative example, consider the development of a predictive model for progression of a rare glomerular disease across multiple institutions, each with limited patient numbers. Using a federated learning framework, participating centers can locally train models on harmonized datasets while sharing only model parameters. The aggregated model benefits from a larger, more diverse dataset without requiring transfer of patient-level data. In practice, such an approach may improve risk stratification and enable earlier identification of patients at risk of rapid progression, although performance remains dependent on data harmonization and consistent implementation across sites.

While centralized aggregation remains the most widely implemented approach, alternative architectures are increasingly being explored to address scalability, robustness, and governance considerations. Decentralized approaches, such as peer-to-peer model exchange, enable institutions to share updates directly without reliance on a single coordinating server, potentially reducing single points of failure and enhancing resilience. Hybrid models combine centralized orchestration with hierarchical or regional aggregation layers, allowing for flexible coordination across institutional networks while preserving local control. These emerging modalities expand the design space for federated learning systems and may be particularly relevant in rare disease contexts, where data are distributed across geographically and organizationally diverse centers.

In practice, operationalizing federated learning requires coordinated computational infrastructure, including secure local environments for on-site model training, reliable communication of encrypted updates, and standardized aggregation procedures. Variability in hardware capacity, network stability, and institutional technical expertise can affect training efficiency, reproducibility, and scalability. In addition, differences in data harmonization, feature representation, and clinical workflow integration may further influence model performance across sites. These considerations highlight that, beyond conceptual advantages, federated learning systems require substantial operational coordination to achieve consistent and clinically meaningful performance across healthcare settings.

### 3.3. The Role of OMOP Common Data Model

Interoperability is the prerequisite for federated AI. The Observational Medical Outcomes Partnership (OMOP) Common Data Model provides standardized tables and vocabularies for demographics, diagnoses, medications, laboratory measurements [72], procedures, and encounters [72,73,74]. Mapping local data to OMOP allows identical training scripts to run across sites, ensuring methodological consistency.

Beyond structural standardization, effective data harmonization requires alignment of coding systems, phenotype definitions, and temporal data representation. This includes mapping local terminologies to standardized vocabularies, normalizing laboratory units, and ensuring consistent representation of longitudinal clinical trajectories. Such harmonization enables models trained across institutions to learn from comparable signals despite differences in local practice patterns and data capture.

### 3.4. Governance and Operational Considerations

Successful multi-institutional AI deployment also depends on robust governance frameworks. These include data use agreements that define permissible data access and model outputs, clear accountability structures across participating institutions, and mechanisms for ongoing model monitoring and validation. Governance must also address issues of bias, fairness, and equitable representation, particularly given the heterogeneity of rare disease populations. In addition, regulatory alignment and auditability are essential to ensure that federated models remain transparent and clinically trustworthy over time [66,67,75,76].

### 3.5. Proof Points Across Domains

Federated learning has already demonstrated feasibility and clinical relevance: [66,67,75,76].

• Multi-institutional imaging and clinical prediction models with performance approaching centralized models while preserving data locality and privacy
• Federated pathogenicity classification models in genomics
• Rare hematologic disease platforms achieving clinically meaningful discrimination
• Federated acute kidney injury prediction across intensive care units

These examples provide direct analogs for rare nephropathies and genetic kidney diseases.

## 4. From Prediction to Individualized Causal Reasoning: Digital Twins in Rare Disease

### 4.1. Biology-Informed Modeling Beyond Statistical Association

An evolving conceptual transition in clinical artificial intelligence increasingly emphasizes biologically informed and individualized modeling approaches rather than systems optimized solely for statistical association [13,14,77]. This perspective is supported by growing literature highlighting the limitations of purely correlation-based models in complex and heterogeneous disease states, particularly in rare diseases where mechanistic priors are strong and data are inherently sparse [78]. Patient-specific digital twin models represent one emerging approach that integrates biological knowledge with patient-specific data to support simulation of disease trajectories and therapeutic responses [13,14].

**Figure 2:**
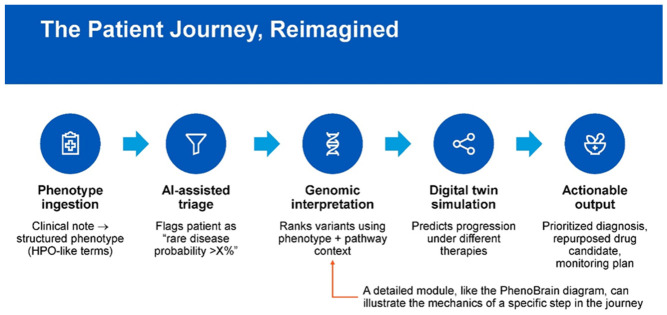
Integrated AI-driven rare disease patient journey. Clinical data are first transformed into structured phenotypes, enabling early AI-assisted triage to identify patients at risk of rare disease. Genomic interpretation integrates phenotype and pathway context to prioritize candidate variants. These outputs feed into patient-specific digital twin models that simulate disease trajectories under alternative therapeutic strategies. The pipeline culminates in actionable clinical outputs, including prioritized diagnoses, therapeutic recommendations, and longitudinal monitoring plans. This figure synthesizes how multiple AI paradigms converge into a workflow-native system for individualized, data-driven care in rare diseases.

In contrast to models that rely primarily on statistical pattern recognition, biology-informed approaches incorporate molecular pathways, genotype–phenotype relationships, and physiological interactions, thereby enabling more interpretable and clinically meaningful predictions [13,14]. This shift from statistical association to mechanism-informed inference is particularly relevant in rare disease settings, where individualized reasoning and causal understanding are essential for accurate diagnosis and treatment planning [13,14]. Figure 2 provides an integrated view of how upstream AI components, including phenotype extraction, triage, and genomic interpretation, combine to support digital twin–based simulation and actionable clinical outputs [78,79,80].

A conceptual clinical application of digital twins can be illustrated in a patient with a rare hereditary nephropathy and variable disease progression. A digital twin model integrating longitudinal laboratory data (e.g., estimated glomerular filtration rate trajectory), genetic findings, and treatment history could simulate disease progression under different therapeutic strategies, such as early versus delayed initiation of renin–angiotensin system blockade. While current implementations remain limited, such models could eventually support individualized treatment planning by providing scenario-based projections, contingent on further validation and clinical integration. This example highlights how digital twin frameworks translate biological and longitudinal data into clinically meaningful simulations, bridging conceptual modeling with potential decision support in rare disease care.

Digital twin models represent an emerging framework for integrating patient-specific data to simulate disease trajectories and therapeutic responses [79,81,82]. While early studies have demonstrated feasibility in selected domains, clinical implementation in rare disease care remains limited [83,84]. Most current applications are based on conceptual models, retrospective analyses, or small-scale studies, and robust prospective or multi-center validation is lacking [83,84]. In addition, variability in data quality, model assumptions, and implementation context further constrains generalizability across clinical settings [77,83]. Accordingly, digital twins should be considered a forward-looking approach with potential for future clinical integration rather than a near-term standard of care, and their adoption will require rigorous validation, standardized evaluation frameworks, and careful integration into clinical workflows.

### 4.2. Architecture of a Rare Disease Digital Twin

A digital twin comprises four integrated layers: [66,77,78,79,85,86].

1. Phenotypic Twin: Structured, time-aware representation of clinical features using standardized ontologies.
2. Molecular and Pathway Twin: Genomic and proteomic representations linking variants to biological mechanisms.
3. Physiology and Organ-System Twin: Functional modeling of organ interactions and disease progression.
4. Intervention Simulation Twin: Counterfactual testing of therapeutic strategies.

Such systems evolve longitudinally as new data accrue, enabling detection of phenotypic drift before clinical thresholds are crossed.

Despite their conceptual appeal, digital twin models face substantial limitations in current practice. Their performance depends on the availability of high-quality, longitudinal, and multimodal data, which are often incomplete, inconsistently captured, or not readily interoperable in real-world clinical settings. In addition, the underlying biological representations may rely on simplified or partially specified models of complex disease processes, potentially limiting predictive accuracy and mechanistic validity. Validation is frequently restricted to small cohorts, retrospective analyses, or simulated environments, and standardized frameworks for evaluating clinical performance remain underdeveloped. These constraints introduce risks of overfitting, misinterpretation, and limited generalizability across patient populations and care settings. Furthermore, the computational complexity and infrastructure requirements of digital twin systems may limit scalability beyond specialized centers. Collectively, these limitations underscore the need for cautious interpretation, rigorous prospective validation, and careful integration into clinical workflows before routine clinical deployment.

### 4.3. Generative AI and Synthetic Patients

Generative models offer a potential solution to data scarcity by synthesizing realistic patient trajectories while preserving statistical properties of real-world data [20,87,88,89,90,91,92]. When rigorously validated, synthetic cohorts may support hypothesis generation, trial design, and algorithm stress-testing in ultra-rare conditions.

Agentic AI systems, which are capable of autonomous task planning and multi-step reasoning, represent a rapidly evolving area of research [93]. By design, these systems can interact with external tools and generate adaptive outputs, increasing both their potential utility and their risk profile. Although early demonstrations in controlled experimental settings are promising, their application in clinical rare disease care remains largely exploratory. Current limitations include susceptibility to reasoning errors, variability in performance across tasks, and limited transparency in decision-making processes. In clinical contexts, these issues raise concerns regarding safety, accountability, and reproducibility. Furthermore, robust validation frameworks for agentic systems remain underdeveloped, particularly in rare disease settings where standardized benchmarks are lacking. Accordingly, these systems should be considered investigational and, if used, deployed within tightly controlled environments with clear human oversight, rather than as independent decision-makers in routine clinical practice.

## 5. Accountability, Regulation, and Adaptive Governance for Rare Disease AI

As rare disease AI systems transition from static models to continuously learning, federated, and agentic architectures, questions of accountability and regulatory oversight become central rather than peripheral. Federated learning environments refer to distributed computational settings in which multiple institutions collaboratively train models using local data, while sharing only model parameters or updates rather than raw patient-level data [94,95,96,97]. In such environments, model performance may drift over time as local data distributions evolve, institutions join or leave networks, or clinical practices change [97,98]. Determining accountability in such settings requires explicit governance structures that define responsibility across stakeholders, including local deploying institutions, central coordinating bodies, and model developers. Without clearly assigned ownership for monitoring, auditing, and corrective action, federated systems risk becoming clinically opaque despite their privacy-preserving design [99,100].

Generative models and synthetic cohorts further complicate this governance landscape. Generative approaches offer a potential solution to data scarcity by synthesizing realistic patient trajectories while preserving statistical properties of real-world data [87,101,102,103,104,105]. Several validated implementations of synthetic clinical data have been reported, demonstrating that appropriately generated datasets can reproduce key statistical distributions and support downstream analytical tasks, including model development and validation [87,106,107]. For example, prior studies have shown that synthetic datasets can approximate real-world clinical quality measures and population-level characteristics, supporting their use in methodological development and benchmarking [87,92,108,109,110,111].

However, these implementations remain context-dependent, and validation is often limited to specific use cases, such as common diseases or structured clinical datasets [87,101,102,103,104,105]. In rare disease settings, where phenotypic heterogeneity is high and sample sizes are extremely limited, the generation of reliable synthetic cohorts remains more challenging and less well established [13,14]. Accordingly, while synthetic data hold promise for hypothesis generation, trial design, and algorithm stress-testing, their clinical applicability requires careful validation to ensure fidelity, generalizability, and absence of bias [87,101,102,103,104,105]. These considerations introduce additional layers of accountability, including the need to define responsibility for synthetic data generation, validation, and downstream use in clinical or research settings.

In rare disease contexts, clinical utility may not be adequately captured by conventional performance metrics such as overall accuracy or area under the curve. Instead, effective systems must demonstrate sensitivity to individual pathogenic signals, defined as the ability to detect clinically meaningful, patient-specific abnormalities, including rare variants or atypical phenotype combinations that may be diluted in population-level analyses. This differs fundamentally from traditional evaluation approaches, which emphasize average performance across large cohorts rather than detection of rare but high-impact signals [20,112,113]. In rare disease settings, leaderboard performance on static benchmark datasets is therefore a limited proxy for clinical utility, which is more appropriately defined by sensitivity to individual pathogenic signals, robustness to data sparsity, and integration into real-world diagnostic workflows [16,45,114,115,116].

The proposed evaluation framework extends beyond conventional metrics by incorporating domains that can be operationalized in practice, including sensitivity to individual pathogenic signals, biological plausibility, and longitudinal consistency. Sensitivity to individual pathogenic signals may be evaluated by assessing the model’s ability to correctly prioritize known pathogenic variants or clinically confirmed diagnoses in patient-specific cases. Biological plausibility can be assessed through concordance with established genotype–phenotype relationships and known disease mechanisms. Longitudinal validation may be operationalized by tracking agreement between predicted and observed disease trajectories over time within the same patient. These domains complement traditional performance measures such as sensitivity, specificity, and area under the curve, which remain important but may not fully capture clinical utility in rare disease contexts. Accordingly, alternative validation frameworks that do not rely solely on randomized controlled trials are required; key validation pillars applicable to rare disease AI are summarized in Table 4 [20,112,113]. These approaches are intended to complement, rather than replace, conventional clinical validation paradigms where feasible.

**Figure 3:**
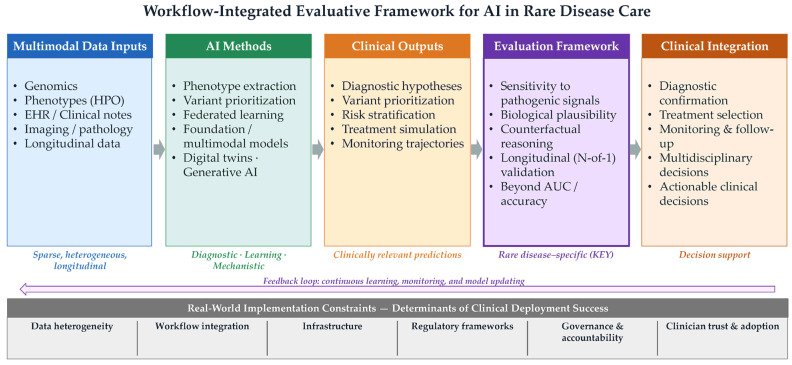
Workflow-integrated evaluative framework for AI in rare disease care. Multimodal data inputs—including genomics, clinical phenotypes, imaging, and electronic health records—are processed through complementary AI approaches such as phenotype extraction, genomic variant prioritization, federated learning, and digital twin modeling. These components converge to generate clinically relevant outputs, including diagnostic hypotheses, risk stratification, and therapeutic insights. Evaluation is structured across multiple domains: sensitivity to individual pathogenic signals, biological plausibility, counterfactual reasoning, and longitudinal validation. The framework also incorporates real-world constraints, including workflow integration, infrastructure requirements, governance, and regulatory oversight, highlighting the pathway from model development to clinically actionable, trustworthy systems. Abbreviations: HPO, Human Phenotype Ontology; AI, artificial intelligence; AUC, area under the curve; N-of-1, within-individual longitudinal evaluation.

Figure 3 provides a workflow-integrated evaluative framework that synthesizes these validation principles with upstream data inputs, AI modeling approaches, and downstream clinical integration. The figure illustrates how rare disease AI systems should be assessed not only on model performance but also on biological plausibility, longitudinal consistency, and real-world deployability within clinical workflows.

Across AI approaches in rare disease care, several cross-cutting limitations warrant careful consideration. Failure modes include false positive prioritization of variants, reduced sensitivity in atypical or incomplete phenotypic presentations, and performance degradation due to data drift over time. Bias may arise from underrepresentation of certain populations in training datasets, resulting in unequal performance across demographic groups and potential disparities in diagnostic accuracy. In addition, models optimized in controlled research environments may not maintain performance when deployed in heterogeneous clinical settings with variable data quality and workflow structures. These challenges underscore the importance of continuous performance monitoring, periodic recalibration, and transparent reporting of model limitations to ensure reliable and equitable use in real-world clinical practice.

Regulatory evaluation of continuously learning AI systems poses additional challenges [117,118,119]. Traditional approval paradigms, which assume fixed algorithms and static performance characteristics, are poorly suited to models that update iteratively or adapt across sites. Emerging regulatory concepts, such as predetermined change control plans and lifecycle-based oversight, offer a potential pathway but remain underdeveloped for federated and rare disease contexts [119,120,121]. In practice, post-deployment surveillance can be implemented through continuous performance monitoring, detection of data drift, and periodic recalibration using local data. Auditability requires maintaining detailed logs of model inputs, outputs, and updates, enabling retrospective evaluation of clinical decisions, while transparency may be supported through model documentation, explainability tools, and clear communication of model limitations to clinicians and patients.

Rare diseases may warrant adaptive regulatory pathways distinct from those applied to common conditions [122,123]. In this context, carefully governed conditional deployment, coupled with rigorous real-world performance monitoring and mandatory reassessment, provides a pragmatic balance between innovation and patient safety [123]. Such frameworks parallel adaptive trial designs, accelerated approval pathways, and regulatory approaches for software as a medical device, offering early access under controlled conditions while ensuring ongoing validation [123,124,125].

Translating AI systems from controlled research environments into routine clinical practice presents substantial challenges. Data heterogeneity across institutions, including differences in documentation practices, coding systems, and patient populations, can significantly affect model performance and generalizability. Integration into clinical workflows requires interoperability with electronic health record systems, user-centered interface design, and alignment with clinician decision-making processes. In addition, implementation demands technical infrastructure, computational resources, and ongoing maintenance that may not be uniformly available. Regulatory uncertainty, particularly for adaptive and continuously learning systems, further complicates deployment. Finally, clinician trust and adoption remain critical barriers, as effective use depends on transparency, interpretability, and demonstrated clinical benefit.

Feasibility of AI deployment varies substantially across healthcare environments [61]. While large academic centers may have access to advanced computational infrastructure, curated datasets, and specialized expertise, smaller or resource-constrained settings may face significant barriers related to data availability, technical capacity, and workforce training. As a result, models developed and validated in highly resourced environments may not directly translate to broader clinical contexts without adaptation. In addition, differences in data standards, interoperability, and clinical workflow integration may further affect performance and usability across settings. Addressing these disparities will require scalable implementation strategies, standardized data frameworks, and targeted efforts to ensure equitable access to AI-enabled care.

In clinical practice, AI-supported decision tools may be used during multidisciplinary case discussions for patients with complex or atypical presentations. For instance, in a patient with discordant biopsy findings and clinical features, AI-assisted summarization of longitudinal data and expansion of differential diagnoses may help identify overlooked rare conditions. Such tools function as decision support systems rather than replacements for clinical judgment, and their effectiveness depends on appropriate integration into existing workflows and clinician oversight.

Taken together, effective governance of rare disease AI will require alignment between technical design, institutional accountability, and regulatory innovation. In practice, this coordination may be operationalized through multidisciplinary oversight committees, standardized reporting frameworks, shared performance benchmarks, and clearly defined stakeholder responsibilities across development, deployment, and monitoring phases. Without such coordination, even technically robust systems risk failing at the point of clinical trust and adoption.

## 6. Conclusions

Current evidence suggests that AI applications may improve diagnostic efficiency, support privacy-preserving multi-institutional discovery, and facilitate the development of patient-specific modeling approaches such as digital twins in rare disease care. Privacy-preserving discovery refers to approaches including federated learning and distributed model training, in which data remain within local institutions while models learn from aggregated, anonymized parameters, thereby facilitating collaborative knowledge generation without direct data sharing. Current evidence suggests that clinically meaningful AI systems in rare disease care should prioritize sensitivity to individual pathogenic signals, interoperability, biological plausibility, and integration into existing clinical workflows rather than focusing solely on incremental gains in benchmark performance metrics. Collectively, these developments position rare disease care as an important translational environment for evaluating clinically integrated AI applications.

However, these advances must be interpreted within the context of important methodological and practical limitations. If validation frameworks remain centered on static datasets, retrospective benchmarks, and population-level metrics, many systems are unlikely to translate effectively into real-world settings, particularly in rare diseases, where delayed or missed diagnoses carry disproportionate clinical consequences. In addition, models optimized primarily for average population-level performance may inadequately capture individualized disease trajectories and heterogeneous phenotypic presentations encountered in rare disease care. Models optimized for average performance rather than individualized reasoning risk reinforcing diagnostic inertia, defined as the persistence of delayed or incomplete diagnostic processes driven by reliance on conventional workflows, anchoring bias, and insufficient integration of longitudinal patient-specific data.

The need for individualized reasoning in rare disease care reflects the inherent heterogeneity and low prevalence of these conditions. In practice, this can be supported by AI systems that integrate structured phenotypic data, genomic information, and longitudinal clinical trajectories to enable N-of-1 inference, phenotype-driven prioritization, and patient-specific risk modeling. Such approaches allow dynamic updating of diagnostic hypotheses and therapeutic strategies as new data become available, aligning AI outputs with real-world clinical decision-making. At the same time, progress in this field will depend not only on algorithmic innovation but also on rigorous validation, careful evaluation of failure modes, and thoughtful integration into clinical workflows. As discussed throughout this review, many emerging approaches, including digital twins, federated learning architectures, and agentic AI systems, remain at varying stages of maturity and clinical validation. Accordingly, their future role in routine practice will depend on prospective evaluation, reproducibility across healthcare environments, and demonstration of clinically meaningful benefit.

While rare disease settings offer a compelling environment for advancing individualized and adaptive AI systems, translation into routine practice requires robust governance, continuous monitoring, and clear delineation of the role of AI as decision support rather than autonomous clinical authority. Overall, the current literature supports cautious optimism regarding the potential of AI-enabled rare disease care while underscoring the continued importance of clinician oversight, implementation science, and real-world translational evaluation. Taken together, a balanced approach that combines innovation with critical appraisal is essential to ensure that artificial intelligence systems deliver reliable, equitable, and clinically meaningful benefits.

## Figures and Tables

**Table 1:** Core Artificial Intelligence Approaches in Rare Disease Care: Mechanisms, Strengths, Limitations, and Clinical Applications. Representative references are provided for scientific transparency and illustrative context.

Approach	Core Concept	Why It Is Relevant in Rare Diseases	Typical Data Sources	Key Strength	Major Limitation	Representative Clinical Applications
Transfer Learning [27]	Reuse knowledge learned from large datasets	Compensates for limited rare disease sample sizes	Imaging, genomics	Strong baseline performance with limited local data	Risk of domain mismatch and reduced generalizability	Rare genetic disorders; rare cancers
Federated Learning [31]	Train models across institutions without sharing raw patient data	Enables multi-center learning while preserving privacy	EHRs, registries	Privacy-preserving scalability	Data heterogeneity and infrastructure complexity	International registries; pediatric rare diseases
Graph Neural Networks (GNNs) [30]	Learn biological relationships within networks and pathways	Captures complex molecular interactions despite small cohorts	Multi-omics, biological networks	Effective with limited sample sizes	Complex graph construction and interpretation	Gene discovery; drug repurposing
Few-shot/Low-resource Learning [27]	Learn from minimal labeled examples	Designed for extreme data scarcity	Imaging, EHRs	Rapid adaptation to small datasets	Sensitivity to noisy or biased labels	Rare phenotype classification
Causal Inference Frameworks [28]	Model potential cause-and-effect relationships	Supports inference when randomized trials are impractical	Longitudinal EHR data	Mechanistic and clinically interpretable insights	Susceptibility to confounding and modeling assumptions	Treatment-effect estimation; drug repurposing
Explainable AI (XAI) [29]	Improve interpretability of model outputs	Enhances clinician trust and auditability	Multimodal	Improved transparency and regulatory readiness	Potential tradeoff with predictive performance	Diagnostics; genomics
Reinforcement Learning (RL) [28]	Optimize sequential clinical decisions over time	Models dynamic disease trajectories and treatment adaptation	Time-series EHR data	Personalized treatment strategies	Requires dense longitudinal data	Treatment sequencing
Foundation/Multimodal Models [30,31]	Integrate text, imaging, genomic, and clinical data	Handles fragmented and heterogeneous rare disease information	Multimodal	Cross-domain integration and generalization	Bias, interpretability, and validation challenges	Diagnostic support; phenotype extraction

Abbreviations: AI, artificial intelligence; EHR, electronic health record; GNNs, graph neural networks; RL, reinforcement learning; XAI, explainable artificial intelligence.

**Table 2:** Comparison of Conventional Variant-Prioritization Pipelines and Genomics Foundation Models in Rare Disease Diagnostics [46,47].

Aspect	Conventional Variant-Prioritization Pipelines (DeepVariant, SpliceAI, AlphaMissense or REVEL, Exomiser)	AlphaGenome (Genomics Foundation Model)
Primary input	Processed sequencing variants	Raw DNA sequence
Modeling approach	Multiple independent task-specific models	Unified sequence-to-biology foundation model
Primary output	Pathogenicity scores and ranked candidate variants	Predicted regulatory, transcriptional, and molecular effects
Coding variant interpretation	Strong performance	Strong performance
Noncoding variant interpretation	Limited coverage	Major strength
Splicing analysis	Separate specialized models (e.g., SpliceAI)	Integrated within unified regulatory predictions
Regulatory element modeling	Limited or indirect	Explicitly modeled
Long-range genomic interactions	Generally absent	Included
Clinical interpretation focus	“Is this variant likely pathogenic?”	“How might this variant alter biological function?”
Typical role in clinical workflow	Variant filtering and phenotype-guided prioritization	Mechanistic interpretation and biological hypothesis generation
Major limitation	Fragmented architecture and limited noncoding interpretation	Reduced interpretability and ongoing need for external clinical validation

Abbreviations: DNA, deoxyribonucleic acid; REVEL, Rare Exome Variant Ensemble Learner.

**Table 3:** Practical Workflow-Integrated AI Applications for Nephrologists Managing Rare Diseases [60,61].

Clinical Scenario	Core Clinical Challenge	AI-Assisted Workflow Application	Potential Clinical Impact
Phenotype summarization at initial evaluation	Rare disease features are dispersed across years of clinical documentation	Summarize longitudinal EHR data into structured phenotype profiles and extract key symptom patterns before genetic evaluation	Shortens diagnostic delays; improves genetics referrals; reduces premature labeling as “idiopathic CKD”
Rare disease detection during routine CKD care	Early disease manifestations are often nonspecific	Identify pattern-based clinical red flags such as hematuria with family history or CKD with extra-renal manifestations	Earlier genetic evaluation; fewer delayed diagnoses at ESKD
Variant interpretation support	Variants of uncertain significance complicate clinical decision-making	Summarize variant annotations and identify clinical features that strengthen or weaken pathogenicity assessment	Supports clinically actionable monitoring and multidisciplinary discussion
Differential diagnosis expansion	Anchoring bias toward common glomerular diseases	Expand differential diagnoses for atypical CKD presentations or discordant biopsy–phenotype findings	Improves rare disease recognition and diagnostic breadth
Clinical trial and expanded-access matching	Dynamic eligibility criteria contribute to missed opportunities	Match evolving phenotypes and laboratory trends to clinical trials or expanded-access programs	Improves trial access and referral equity
Longitudinal disease monitoring	Disease progression may occur gradually over years	Track longitudinal trajectories including eGFR decline, proteinuria trends, and evolving symptom patterns	Earlier intervention and more individualized treatment escalation
Patient communication and education	Rare disease counseling is often inconsistent and time-intensive	Generate patient-specific educational summaries aligned with diagnosis, laboratory findings, and treatment plans	Improves adherence, communication efficiency, and shared decision-making
Multidisciplinary case preparation	Multidisciplinary reviews are labor-intensive	Prepare structured case summaries including phenotype data, genomic findings, unresolved questions, and management considerations	More efficient multidisciplinary discussions and standardized documentation

Abbreviations: AI, artificial intelligence; CKD, chronic kidney disease; EHR, electronic health record; ESKD, end-stage kidney disease; eGFR, estimated glomerular filtration rate.

**Table 4:** Validation Approaches for AI Systems in Rare Disease Care Beyond Conventional Randomized Trials.

Validation Domain	Core Clinical Question	What Is Evaluated	Relevance in Rare Diseases	Comparison with Conventional Validation
Causal plausibility	Does the model output align with known disease biology?	Concordance between AI predictions and established genotype–phenotype or pathway relationships	Rare diseases often have strong mechanistic foundations that support biologically informed validation	Complements population-level statistical inference
Counterfactual simulation	Could an alternative intervention plausibly alter patient outcomes?	Simulation of disease trajectories under different therapeutic strategies within patient-specific models	Supports individualized causal reasoning when randomized trials are impractical or infeasible	Complements parallel control-arm comparisons
Longitudinal N-of-1 validation	Does the prediction remain consistent over time within the same patient?	Agreement between predicted and observed longitudinal trajectories including laboratory values, imaging findings, and symptoms	Particularly relevant in slowly progressive and highly individualized rare diseases	Complements large cohort reproducibility assessments

Abbreviations: N-of-1, single-patient (within-individual) longitudinal evaluation.

## Data Availability

No new data were created or analyzed in this study.

## Abbreviations

The following abbreviations are used in this manuscript:

AI	Artificial intelligence
ACEi	Angiotensin-converting enzyme inhibitor
CKD	Chronic kidney disease
*COL4A*	Type IV collagen alpha chain genes
EHR	Electronic health record
ESKD	End-stage kidney disease
GNNs	Graph neural networks
GBM	Glomerular basement membrane
HPO	Human Phenotype Ontology
MDT	Multidisciplinary Team
REVEL	Rare Exome Variant Ensemble Learner
RL	Reinforcement learning
RCTs	Randomized controlled trials
VUS	Variant of Uncertain Significance
XAI	Explainable artificial intelligence
